# Prediction of LDL cholesterol response to statin using transcriptomic and genetic variation

**DOI:** 10.1186/s13059-014-0460-9

**Published:** 2014-09-30

**Authors:** Kyungpil Kim, Eugene Bolotin, Elizabeth Theusch, Haiyan Huang, Marisa W Medina, Ronald M Krauss

**Affiliations:** Children’s Hospital Oakland Research Institute, 5700 Martin Luther King Jr Way, Oakland, CA 94609 USA; Department of Statistics, University of California, Berkeley, CA 94720 USA

## Abstract

**Background:**

Statins are widely prescribed for lowering LDL-cholesterol (LDLC) levels and risk of cardiovascular disease. There is, however, substantial inter-individual variation in the magnitude of statin-induced LDLC reduction. To date, analysis of individual DNA sequence variants has explained only a small proportion of this variability. The present study was aimed at assessing whether transcriptomic analyses could be used to identify additional genetic contributions to inter-individual differences in statin efficacy.

**Results:**

Using expression array data from immortalized lymphoblastoid cell lines derived from 372 participants of the Cholesterol and Pharmacogenetics clinical trial, we identify 100 signature genes differentiating high versus low statin responders. A radial-basis support vector machine prediction model of these signature genes explains 12.3% of the variance in statin-mediated LDLC change. Addition of SNPs either associated with expression levels of the signature genes (eQTLs) or previously reported to be associated with statin response in genome-wide association studies results in a combined model that predicts 15.0% of the variance. Notably, a model of the signature gene associated eQTLs alone explains up to 17.2% of the variance in the tails of a separate subset of the Cholesterol and Pharmacogenetics population. Furthermore, using a support vector machine classification model, we classify the most extreme 15% of high and low responders with high accuracy.

**Conclusions:**

These results demonstrate that transcriptomic information can explain a substantial proportion of the variance in LDLC response to statin treatment, and suggest that this may provide a framework for identifying novel pathways that influence cholesterol metabolism.

**Electronic supplementary material:**

The online version of this article (doi:10.1186/s13059-014-0460-9) contains supplementary material, which is available to authorized users.

## Background

Statins reduce low density lipoprotein cholesterol (LDLC) levels by inhibiting 3-hydroxy-3-methylglutaryl coenzyme A reductase (*HMGCR*), the enzyme that catalyzes the rate-limiting step of cholesterol biosynthesis. Although numerous clinical trials have demonstrated statin efficacy for reducing cardiovascular disease risk, and have shown that this is proportional to LDLC lowering [1], there is substantial variation among individuals in the magnitude of this response [2,3].

Variation in the LDLC response to statin treatment has been attributed to both phenotypic and genetic factors. Phenotypic predictors include age, ancestry and smoking status [2]. Genetic association studies have also identified some single nucleotide polymorphisms (SNPs) and haplotypes associated with statin response [4-8]. For example, candidate gene analyses have found variants in known regulators of cholesterol metabolism such as *HMGCR*, *APOE*, *PCSK9*, *ACE*, and *LDLR* to be associated with statin response [5-7]. In addition, genome-wide association studies (GWAS) have identified several SNPs in the *LPA* and *APOE*/*TOMM40* loci that achieved genome-wide significance for association with the magnitude of LDLC reduction [9]. However, taken together, these genotypes account for only a small proportion of the variation (approximately 4%) in statin-mediated LDLC reduction [9]. On the other hand, alternative splicing of *HMGCR* in lymphoblastoid cell lines (LCLs) was found to explain >6% of the variance in LDLC response in individuals from whom the LCLs were derived [10]. Notably, rs3846662, a SNP that directly regulates *HMGCR* alternative splicing, in itself was not a significant determinant of statin response, demonstrating that investigation of variation at the level of the transcriptome may be more powerful for detecting novel markers of statin efficacy compared to traditional SNP association studies.

Gene expression profiling of patient-derived cell lines has been used to identify a panel of genes, or signature genes, associated with response to various drugs [11]. In the present study we sought to identify a transcriptomic profile associated with variation in LDLC response to statin treatment using non-negative matrix factorization (NMF) and radial-basis support vector machines (SVMs) prediction models to define a panel of signature genes whose expression levels differed between extremes of the LDLC response distribution. We then further refined our prediction model by incorporating SNPs either associated with expression levels of the signature genes (eQTLs) or previously associated with statin response by GWAS. Our present study represents the first attempt to predict inter-individual variation in LDLC response to statin treatment using both transcriptomic and genomic information.

## Results

### Identification of signature genes characterizing high and low statin responders

NMF is a useful feature extraction tool for multivariate data. It attempts to decompose the input data into a product of two non-negative matrices (that is, non-negative basis vectors and coefficients) to represent the data in a low dimensional feature space [12,13]. NMF has been successfully used to distinguish cancer subtypes based on large-scale and genome-wide gene expression data [14-16].

Using transcriptomic data of LCLs derived from 372 Caucasian non-smoking participants of the Cholesterol and Pharmacogenetics (CAP) simvastatin clinical trial (ClinicalTrials.gov ID: NCT00451828, Table 1) [17], we performed NMF clustering to determine the optimal number of individuals defined as either ‘high’ *or* ‘low’ responders in the age-adjusted LDLC response distribution curve (Additional file 1: Figure S1).

**Table 1:** **Baseline clinical characteristics of participants in our study**

	***CAP372***	***CAP212***	***P*** **value**
N	372	212	-
Men	54%	52%	0.62
Smoker (%)	0%	36%	2.2 × 10^-6^
Age (years)	54.9 ± 12.6	53.7 ± 12.6	0.29
BMI (kg/m^2^)	27.7 ± 5.3	27.7 ± 5.3	0.89
LDLC level (mg/dl)	132 ± 28	134 ± 37	0.38
LDLC level change after statin treatment (mg/dl)	-56.7 ± 19.8	-55.7 ± 23.8	0.61

Data are presented as numbers, percentages or means ± SDs.

BMI: Body mass index.

**Figure 1:**
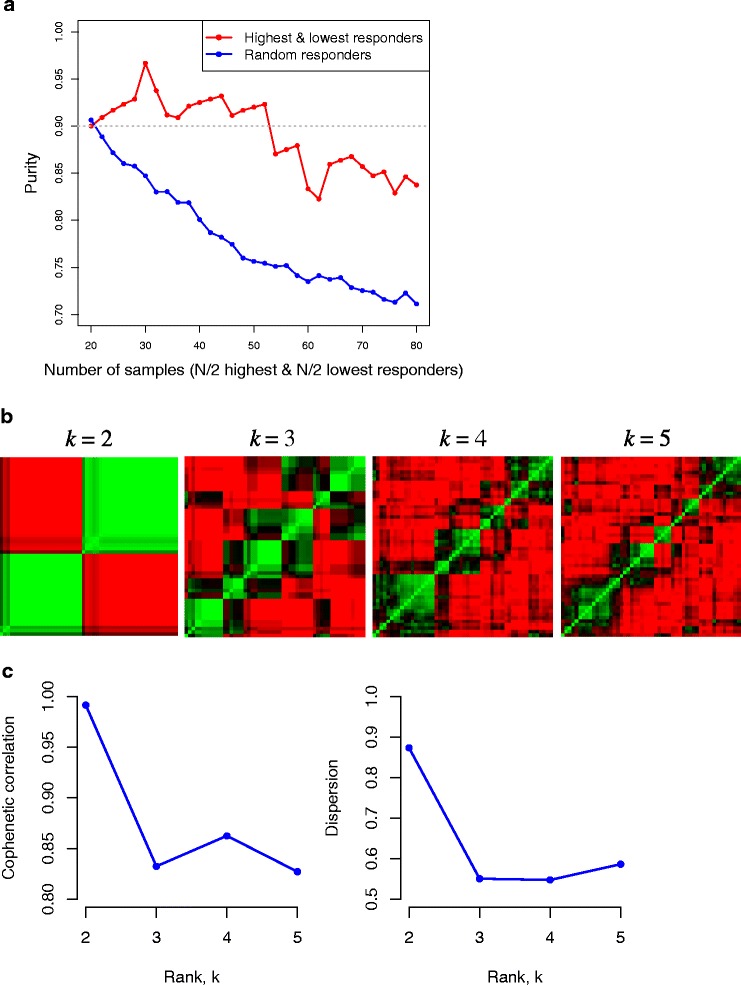
**Summary of the NMF clustering results. (a)** *Purity* curves measuring the performance of NMF in clustering. The red line was calculated from the *N*/2 highest and *N*/2 lowest samples (*N* = 20, …, 80) and the blue line was obtained from the *N* randomly selected samples from the entire set of samples. *Purity* cutoff, 0.9, is denoted with a grey dotted line. **(b)** Consensus matrices for the 26 highest and 26 lowest responder samples, averaging 500 connectivity matrices computed at *k* = 2, 3, 4, 5. Comparison of the visualized consensus matrices across different ranks is a graphical way of deciding the best rank since clear block patterns along the diagonal of the consensus matrices indicate robustness of clustering in the corresponding ranks. **(c)** Cophenetic correlation and dispersion measures assessing the stability of clustering associated with each rank *k*.

We evaluated sample numbers ranging from 20 to 80 (with samples evenly divided between the extremes of the high and low response tails), by progressively including samples from less extreme ranges of the response distribution. A similar analysis of two randomly selected groups was also performed for comparison of separation to the true high *versus* low response groups. To maximize the *purity* difference between the true high *versus* low responder groups and the randomly selected group, as well as maximize the purity while maintaining a reasonable sample size for subsequent analyses, we selected 52 samples, 26 from each responder group (Figure 1a; Additional file 1: Figure S2). We found that expression data from these samples had the most robust clustering when divided into two groups (or ranks), compared to three, four, or five groups (Figure 1b and c). Stable clustering into two groups indicates that the tails of the LDLC change distribution are discrete sets, and that individuals could be categorized into high and low response groups by gene expression measures alone.

**Figure 2:**
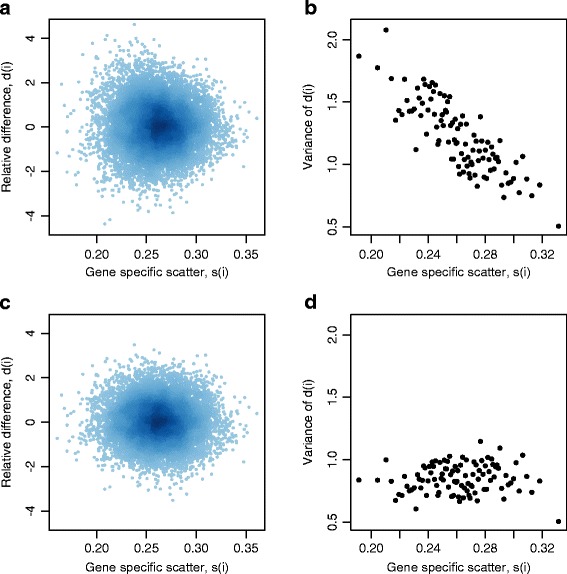
**Gene specific scatter plots.** Scatter plots of the gene specific scatter *s*(*i*) *vs.* the relative difference *d*(*i*) with **(a)** *s*_0_ = 0 and **(c)** the varying *s*_0_ values. The corresponding variance of *d*(*i*) as *s*(*i*) increases is shown in **(b)** and **(d)**, respectively.

**Figure 3:**
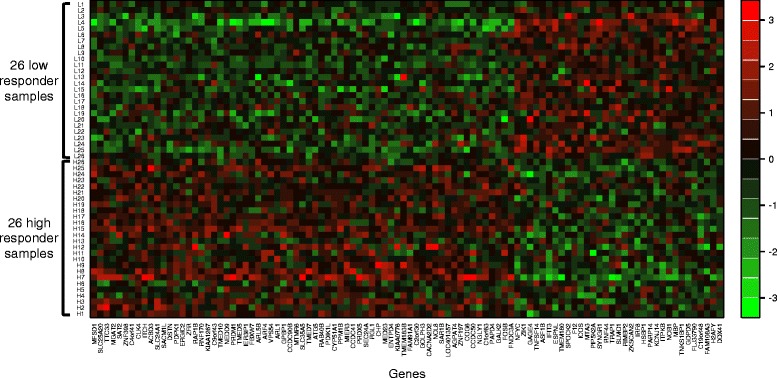
**Heatmap comparing expression levels of the 100 genes in** ***SG*** **between high** ***vs.*** **low responders.** Each column corresponds to a gene, and each row corresponds to a sample: H1 to H26 are high responders and L1 to L26 are low responders. Expression levels for each gene were normalized as described in the Materials and methods section. Expression levels greater than 0 are shaded in red, and those less than 0 in green.

Next, we designed an algorithm to identify differentially expressed genes between the selected high and low responders. Specifically, we adapted a version of empirical Bayes moderated *t*-statistics to test differential expression. A *t*-statistic for each gene *i* is the ratio of the average gene expression difference of the high and low responders and the sample standard error. To obtain reliable estimates of sample standard errors and reliable *t*-tests, empirical Bayes methods have been popularly used to shrink the gene-wise sample variances towards a common value. Following a similar idea, Tusher et al. [18] introduced a constant *s*_0_ (that is related to a prior expected value of standard error *s*(*i*) and proposed to use *s*(*i*) + *s*_0_ as an estimate of the gene-wise sample standard error, resulting in a moderated statistic *d*(*i*). However, we found that this *d*(*i*) was still sensitive to the expression level of each gene and presented increasing variability when *s*(*i*) decreases (Figure 2a and b). To further stabilize the variance of *d*(*i*), we introduced varying *s*_0_ values to the standard error estimates, with results shown in Figure 2c and d. Genes were then ranked based on the statistical significance of the corresponding test statistics (details are in Materials and methods), and the top 100 most significant genes were selected based on their prediction performance compared to other gene sets with smaller or larger numbers of genes (Additional file 1: Figure S3). For ease of notation, these 100 genes were denoted as *SG*, and another set of genes obtained in the absence of *s*_0_ was denoted as *SG*_*NO*_. Of the 100 genes in *SG*, 67 were expressed to a greater extent in high responders than low responders while the other 33 genes were more highly expressed in low responders (Figure 3; Additional file 1: Table S1).

Two of the 100 *SG* genes identified, cytochrome P450, family 51, subfamily A, polypeptide 1 (*CYP51A1*) and nuclear transcription factor Y, gamma (*NFYC*), have known roles in cholesterol metabolism [19,20]. In addition, 48 of the 100 genes were significantly enriched among 3,170 *HMGCR* correlated genes (FDR adjusted *P* <0.05), and this degree of enrichment was substantially greater than expected by chance (*P* <1.3 × 10^-4^). *HMGCR* encodes the rate-limiting step of the cholesterol biosynthesis pathway, and expression levels of this gene can be used as a quantitative marker of cellular cholesterol content [21]. Furthermore, using the enrichment analysis tool *Enrichr* [22], we found that miR-200B and miR-429 predicted binding sites were over-represented in the *SG* list (FDR adjusted *P* = 4.5 × 10^-3^). These micro-RNAs have been previously described to target both *SREBP-1c* and *HMGCR* [23]. Thus these results are consistent with the possibility that other genes identified within the *SG* list may impact cholesterol metabolism.

### Prediction using *SG*-based models

**Figure 4:**
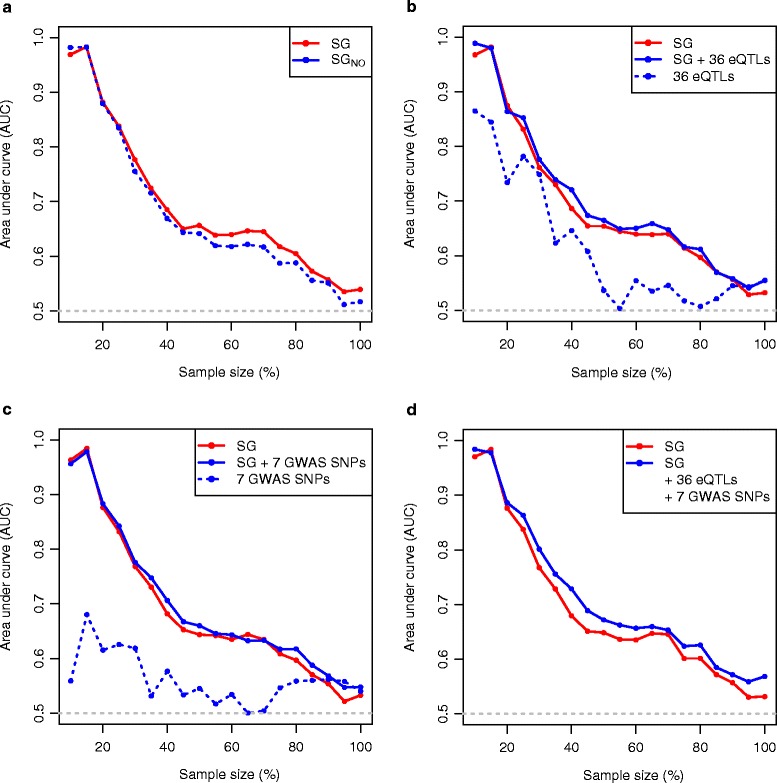
**Prediction performance of classification models incorporating** ***SG*** **expression levels and additional genetic features.** AUC plots, calculated from the corresponding ROC curves, from the prediction models, incorporating various features or a combinations of **(a)** *SG* and *SG*_*NO*_, **(b)** *SG* and 36 eQTLs, **(c)** *SG* and 7 GWAS SNPs, **(d)** all of the features.

SVMs and related kernel methods are extremely good at solving prediction problems in computational biology such as prediction of a gene’s function, its interactions, and its role in disease. This is due to the ability of these methods to deal with high-dimensional datasets, their flexibility in modelling diverse sources of data, and, most importantly, their ability to capture any complex relationships that may exist between different measurements [24,25]. Using radial-basis SVMs, we constructed and compared several different predictive models, each based on transcriptomic features, genotypic features, or a combination of these, for the purpose of classification and regression. In the classification model, we trained and predicted the tails from the LDLC change distribution as binary values (‘high’ or ‘low’ responders). To evaluate classification performance, we conducted receiver operating characteristic (ROC) analysis and reported the area under the curve (AUC) (Figure 4; Additional file 1: Figure S4). In the regression model, we trained and predicted statin-mediated LDLC change as a continuous variable from the full study population to estimate the explained variance of statin-mediated LDLC change.

First we built a classification model based on the expression levels of *SG*. Prediction performance was evaluated by cross-validation as described in ‘Materials and methods’, starting from a sample size of 10% of the population (5% each of the highest and lowest responders), and iteratively increasing the sample number by 5%. The AUC of the model reached a maximum of 0.98 with 15% of the tails and then gradually decreased to 0.54 with the complete dataset (Figure 4a). This result demonstrates that *SG* has more power to predict the extreme responders and less power to predict statin response across the entire study population. We also compared the prediction performance of *SG*_*NO*_ with *SG* (Figure 4a), and found that the *SG*-based model had improved power across all sample sizes.

Using the regression model, the *SG*-based model explained 12.3% of the variance in statin-induced change in LDLC (Table 2). We further tested the variance explained by the *SG*-based model for other plasma lipid phenotypes including baseline LDLC and statin-induced changes in triglyceride, apolipoprotein B (APOB), and high-density lipoprotein cholesterol (HDLC). The explained variance in these phenotypes was negligible, indicating that *SG* is specifically related to LDLC change.

**Table 2:** **Explained variance in plasma lipids of the entire** ***CAP372*** **by signature gene expression levels of** ***SG***

	**Explained variance (%)**
Change in LDLC	12.3
Baseline LDLC	0.8
Change in triglyceride	1.9
Change in APOB	0.3
Change in HDLC	0.2

### Prediction and validation using eQTL SNPs

To determine if incorporation of other genetic features might improve our model, we used five publicly available datasets (Additional file 1: Table S2) to identify SNPs associated with expression levels (eQTLs) of the *SG* genes at *P* <5 × 10^-8^. From a total of 3,317 eQTLs associated with expression levels of 36 *SG* genes, we selected the most strongly associated SNP for each gene (Additional file 1: Table S3).

The AUC plot from a model that only included the 36 eQTL genotypes had a shape similar to that obtained using the *SG*-based model, although not unexpectedly, overall prediction performance was decreased (Figure 4b). Similar to the *SG*-based model, the 36 eQTL based model also exhibited higher prediction power for the extreme responders, with the AUC of 0.86 for the 10% tails and 0.75 for the 30% tails. When we included the 36 eQTLs in the *SG*-based model, such that 36 genes were represented by both their expression levels and their best eQTL genotypes, while the remaining 64 genes were represented by their expression level measurements alone, the overall prediction performance improved slightly and the AUC for the very extreme responders (10% tails) increased from 0.96 to 0.99. The combined model with *SG* and 36 eQTLs explained 13.5% of variance in change of LDLC, which was 1.2% higher than the variance explained by the *SG* based model.

Since the improvement caused by the incorporation of eQTLs into the *SG*-based model was modest, we hypothesized that the eQTLs were highly redundant with the expression levels of the *SG*. Confirming our hypothesis, when the 36 signature genes’ expression levels were replaced by the 36 eQTLs in the *SG* model (64 signature genes’ expression levels + 36 eQTLs), the prediction curve did not change significantly (data not shown), with 12.2% of the variance explained *vs.* 12.3% for the *SG*-based model. However, the sum of the variance explained by the 64 signature genes alone (9.5%) and the 36 eQTLs alone (1.3%) was less than that for the combined model (10.8% *vs.* 12.2%) suggesting a synergistic interaction between these two feature sets.

Notably, we found that the ability of eQTLs to substitute for signature genes was highly dependent on the significance of eQTL *P* values. For example, when 56 eQTLs less strongly correlated with expression levels of the *SG* genes (*P* <10^-5^) replaced the corresponding 56 genes in the *SG*-based model, the model explained 1.5% less variance in LDLC change compared to the *SG*-based model.

Although the CAP trial included 584 self-identified Caucasians with genome-wide genotype data, only 372 subjects were used in the initial analysis due to either smoking status (*N* = 64) or lack of gene expression data (*N* = 148). The remaining subgroup of 212 individuals, designated *CAP212*, was very similar to our initial study population (*CAP372*) in demographic and clinical characteristics with the exception of smoking status (Table 1). In addition, although we previously reported that smoking status is associated with variation in statin-induced LDLC change [2], we did not observe this relationship in the self-reported Caucasian subgroup, indicating that *CAP212* may be an appropriate population to investigate the utility of the 36 *SG* eQTLs as genetic markers for discriminating high and low statin responders. As shown in Table 3, using samples comprising the 15%, 20%, and 25% tails of the LDLC response distribution, the variance explained by the 36 *SG* eQTLs-based model for *CAP212* was 17.2%, 14.6%, and 6.4% while the respective values were 8.2%, 8.5%, and 13.4% for *CAP372* and 6.7%, 7.0%, and 6.1% for the full CAP population (*CAP584*).

**Table 3:** **Explained variance (%) in statin-mediated LDLC reduction calculated by 36 eQTLs based prediction model**

	**Sample size (%)**		
	**15**	**20**	**25**
*CAP212*	17.2	14.6	6.4
*CAP372*	8.2	8.5	13.4
*CAP584*	6.7	7.0	6.1

### Prediction using additional genetic features

We next tested the extent to which individuals in the extreme tails of the LDLC response distributions could be discriminated by a model including SNPs previously reported in GWAS studies to be associated with the magnitude of LDLC reduction at a genome-wide level of significance [9,26]. Seven SNPs were identified that were previously annotated as representing the *APOE*, *ABCG2,* and *LPA* genes (Additional file 1: Table S4). As shown in Figure 4c, the AUC plot for the model based on the seven GWAS SNPs did not exhibit substantial prediction power for the extreme responders. This suggests that genetic markers strongly related to statin response in the entire population are not necessarily informative for discriminating the very high and low responders [27]. When the GWAS SNPs were added to the *SG*-based model, prediction performance was unchanged for the extreme responders but slightly increased when the middle responders were considered as well. This combined model explained 13.8% of the variance in statin-induced change in LDLC, which was comparable to the amount of variance explained by the *SG* with the 36 eQTLs-based model (13.5%).

To determine if addition of SNPs with sub-genome-wide associations (*P* <10^-6^) further improved our model, we incorporated 15 such SNPs (for a total of 22 SNPs) [9,26]. Although the 22 SNPs-based model slightly improved the prediction for the extreme responders, the overall prediction performance was decreased (Additional file 1: Figure S5), suggesting that only SNPs that achieve genome-wide significance are capable of improving predictive power.

Finally we combined all the features into one model including *SG*, eQTLs, and GWAS SNPs (Figure 4d). Compared to the *SG*-based model, the prediction performance increased substantially across all sample sizes and explained 15.0% of the variance in LDLC change, 2.7% more than the *SG-*based model alone. Although no prominent synergistic interaction was observed among different features, inclusion of these additional genetic features substantially increased the explained variance in statin efficacy compared to gene expression levels alone.

## Discussion

Obtaining a comprehensive understanding of genetic factors contributing to variation in drug response is critical for understanding the molecular pathways underlying these differences and providing tools for optimizing treatment for individual patients. For statins, candidate gene and genome-wide SNP association studies have identified only a few loci associated with variation in statin response as assessed by reduction in LDLC, and together, account for only a small portion of variance in LDLC response. To find additional genetic predictors of statin efficacy, we first tried to identify signature genes using an empirical Bayes moderated *t*-statistic that searches for differentially expressed genes between high and low responders by properly shrinking the gene-wise sample variances. Using prediction models based on radial-basis SVMs, we demonstrated that the variation from the signature genes was able to differentiate ‘high’ *versus* ‘low’ statin responders. We then refined our prediction model by including SNPs either associated with expression levels of the identified signature genes (eQTLs) or directly associated with statin response in previously published GWAS analyses. Overall our approach accounted for 15.0% of the variance of LDLC response to simvastatin. To our knowledge this is the largest proportion of variance in statin efficacy explained by molecular biomarkers to date.

Two of 100 signature genes, *CYP51A1* and *NFYC*, have been previously implicated in cholesterol metabolism. The enzyme encoded by *CYP51A1*, lanosterol 14-alpha-demethylase, catalyzes the conversion of lanosterol to 24,25-dihydrolanosterol in one of the later steps of the cholesterol biosynthesis pathway. Interestingly, we found that ‘high’ statin responders had greater levels of endogenous *CYP51A1* gene expression compared to ‘low’ responders, consistent with the possibility that endogenously high levels of cholesterol synthesis may result in greater LDLC lowering with statin treatment. Of our signature gene list that differentiates ‘high’ *versus* ‘low’ responders, *NFYC* is the most highly expressed gene in the ‘low’ responder group (Additional file 1: Table S1). *NFYC* encodes one subunit of the NFY trimeric complex comprised of *NFYA*, *NFYB,* and *NFYC*, which functions as a transcription factor [19,28]. NFY has been shown to interact with sterol regulatory element binding transcription factor 2 (*SREBF2*, aka *SREBP2*), the major transcription factor known to modulate both the cholesterol synthesis and uptake pathways to maintain intracellular cholesterol homeostasis [29]. Interestingly, *NFYC* was very recently identified to be a target of mir-33* [19], the passenger strand micro-RNA generated during the expression of mir-33, a well-established regulator of lipid homeostasis [28].

Since *HMGCR* encodes the rate-limiting enzyme in the cholesterol biosynthesis pathway, and its transcript levels are very precisely regulated [30], variation in *HMGCR* expression can be considered to be a marker of intracellular cholesterol homeostasis. Our findings that expression levels of almost half of the *SG* genes were correlated with expression of *HMGCR,* and that the *SG* genes have an over-representation in miR-200B and miR-429 predicted binding sites, are consistent with the likelihood that these genes are regulated by changes in intracellular cholesterol levels and thus may impact cholesterol metabolism. Since the majority of the *SG* genes have not been previously implicated in cholesterol metabolism, functional studies will be necessary to determine if these genes are not only markers, but also determinants of cellular cholesterol metabolism.

Substituting gene expression levels with eQTLs is a new and promising approach for the development of diagnostic tools, as novel eQTLs are rapidly discovered from various tissues [31]. We found that when the eQTLs were very strongly correlated with gene expression variation (*P* <5 × 10^-8^), replacement of the gene expression measurements with the SNP genotypes had little detrimental effect on the predictive ability of our model. These results suggest that genotype information of robustly associated eQTLs can largely substitute for gene expression data, and hence that analyses of transcript expression levels can serve as a proxy for underlying genetic variation. Not unexpectedly, we found that as the association between gene expression levels and SNP genotype lessened, the ability of eQTLs to substitute for gene expression was diminished.

While replication in an independent population is typically the gold standard used for validating genetic associations, our particular population is unique in that we have measures of *in vivo* statin response from participants of a clinical trial paired with measures of cellular gene expression from LCLs established from those participants. Since we had not utilized data from the entire CAP population and to our knowledge there is no other similar dataset or cell repository, given the ability of the eQTLs to replace gene expression measurements, we attempted to ‘replicate’ our results by testing only the effect of the eQTLs model to predict statin response in the *CAP212* population. Unexpectedly, we found that the model appeared to have even better predictive power in defining the extreme (<20%) tails than the *CAP372* subset of the population that was used to originally define the model, consistent with the likelihood that the *SG* are related to variation in statin response. Notably, the *CAP212* subset has a greater representation of extreme low responders compared to the *CAP372* subset (Additional file 1: Figure S6). Thus, the increased predictive power of the eQTL model in the *CAP212* subset is consistent with our observations that the models have greater power at the tails of the distribution. However, replication should be tested in additional relevant populations as they become available.

When we assessed the power of SNPs identified by GWAS to predict statin response, unlike the *SG* genes or eQTLs, we found that they were less informative for predicting the extreme responders. This performance discrepancy may be due to the fact that eQTLs in our study were derived from genes that reflect only variation between the extreme responders of the population while GWAS SNPs were discovered from more general populations.

## Conclusions

We propose novel integrated prediction models to investigate inter-individual variation in statin efficacy using a comprehensive method that combines transcriptomic and genetic information. With this approach, we explain a substantial percentage of variation in LDLC response to simvastatin treatment. As genotypic, eQTLs and phenotypic datasets grow, our approach can provide a promising framework for identifying novel genes, SNPs, and pathways involved in drug response.

## Materials and methods

### CAP study participants

The Cholesterol and Pharmacogenetics (CAP) trial involved 944 healthy volunteers (609 self-identified Caucasians) selected on the basis of plasma cholesterol levels between 160 and 400 mg/dL who were treated with 40 mg/day simvastatin for 6 weeks. The CAP clinical trial (NCT00451828) was approved by IRBs at the San Francisco General Hospital (H11082-19536), UCLA School of Medicine (01-11-089), where recruitment was performed, as well as the Children’s Hospital Oakland Research Institute (2002-032), where the study was coordinated. All participants provided informed consent before enrollment, and research was carried out in accordance with the Helsinki declaration. Fasting plasma was collected at two pre-treatment time points (screening visit and enrollment visit) and at two post-treatment time points (4 and 6 weeks of treatment). Because LDLC levels were not significantly different between screening and enrollment, the average of these two measurements was used as the pre-treatment LDLC value to minimize technical variation. For the same reason, the average of 4- and 6-week measurements was used as the post-treatment LDLC value. Since the absolute change in LDLC is very highly correlated with the baseline values in the LDLC, we performed analyses on the relative change in LDLC, *log*(*LDLC-change*), which was defined as *log*(post-treatment LDLC value) - *log*(pre-treatment LDLC value) (Additional file 1: Figure S7). To adjust for the clinical covariate effects, three candidate covariates (sex, age, and BMI) were tested with linear regression but only the age covariate significantly related to change in LDLC (*P* <6.6 × 10^-4^) was adjusted. The distribution of the adjusted change measure, *log*(*LDLC-change*), is shown in Additional file 1: Figure S1.

### Gene expression measurements

Gene expression levels were measured using the Illumina Human-Ref8v3 beadarray in 480 LCLs derived from Caucasian American participants in the CAP study, after 24 h incubation under standardized conditions as previously described [17]. We excluded smokers (64 samples) as well as 44 samples for which, after using a Bayesian method of predicting SNP genotypes from expression data of the LCLs, the predicted genotypes did not accurately match directly measured genotypes from the plasma of the LCL donor [32]. The baseline characteristics of the remaining 372 CAP participants (*CAP372*) are presented in Table 1. Each array was quantile transformed to the overall average empirical distribution across all arrays. Expression levels of each gene were then quantile normalized, adjusted for known covariates (date, RNA labeling batch, beadarray hybridization batch, and gender) and quantile normalized again.

### Genotype data

Genotyping was performed on the HumanHap300 (*N* = 304) or HumanHap610-Quad (*N* = 280) BeadChips (Illumina, San Diego, CA, USA) as previously described [4]. These genotypes were imputed to over 2 million SNPs using BIMBAM [33,34] and HapMap CEU as a reference as previously described [4].

### Selecting subsets of the high and low responders using NMF analysis

NMF aims to extract a small number of features (*k*), each defined as a positive linear combination of *n* genes, and express gene expression level as a positive linear combination of these pre-defined features. Given a non-negative data matrix **A**_*n×m*_ (*n*: number of gene, *m*: number of samples), NMF factorizes **A** into two matrices **W**_*n×k*_ and **H**_*k×m*_. Each of the *m* columns in **H** matrix represents the predicted class expression pattern of the corresponding sample, which is used to assign *m* samples into *k* predicted classes. The stochastic nature of the seeding method used to generate the initial **W** and **H** matrices requires multiple NMF runs to achieve stability and the lowest approximation error. For this purpose, we used the NMF R package [35] and all the results from NMF analysis reported in this work were based on the best fit after performing 500 runs for each dataset. To meet the non-negative requirement of the data matrix, each entry in the normalized gene expression data matrix was replaced with the value of its *P* value subtracted from one, where the *P* values were calculated under the assumption of normality.

In order to select subsets that reflect the biological distinctions between high and low responder groups, NMF clustering was used with the following strategy fixing the number of features or ranks to two so as to represent the two extreme responder groups: (1) choose *N/2* (10 to 40) each of the highest and lowest responder samples, *N* in total; (2) select 2,000 genes that have the greatest mean difference between the two groups. Two thousand was the maximum number of genes that provided separation between two groups in our study; (3) Perform NMF analysis and evaluate cluster quality using the *purity* and *entropy* measures; (4) repeat (1) to (3) for another randomly selected 500 sets of *N* samples and calculate the average *purity* and *entropy* values for comparison. To evaluate the cluster quality of NMF analysis, *purity* and *entropy* measures were used [15]. *Purity* is a measure of cluster coherence while *entropy* measures how the various classes of samples are distributed in a cluster. Given *l* true class labels, suppose NMF generates *k* clusters. *Purity* is defined as $\frac{1}{n}{\displaystyle {\sum}_{i=1}^k\underset{1\le j\le l}{ \max}\left({n}_i^j\right),}$ where *n* is the total number of samples and ${n}_i^j$ is the number of samples in the cluster *i* that belong to original class *j. Entropy* is given by $-\frac{1}{n \log {}_2l}{\displaystyle {\sum}_{i=1}^k{\displaystyle {\sum}_{j=1}^l{n}_i^j \log {}_2\frac{n_i^j}{n_i}}},$ where *n*_*i*_ is the size of cluster *i. Purity* values close to 1, and *entropy* values close to 0 represent perfect clustering.

### Identifying signature genes

To identify signature genes from the subsets selected, we first defined a score *relative difference* (in the same form as a *t*-statistic), *d*(*i*), for each gene on the basis of change in gene expression relative to the *gene specific scatter*, *s*(*i*), which is the standard deviation of repeated measurements. In particular, the *relative difference* and the *gene specific scatter* are defined as

1 $$ d(i)=\frac{{\overline{x}}_H(i)-{\overline{x}}_L(i)}{s(i)+{s}_0} $$

2 $$ s(i)=\sqrt{\frac{1}{N\left(N-1\right)}\left({\displaystyle {\sum}_{H=1}^N{\left[{x}_H(i)-{\overline{x}}_H(i)\right]}^2}+{\displaystyle {\sum}_{L=1}^N{\left[{x}_L(i)-{\overline{x}}_L(i)\right]}^2}\right)} $$

where ${\overline{x}}_H(i)$ and ${\overline{x}}_L(i)$ are defined as the average levels of expression for gene *i* in the high (*H*) and low (*L*) responder groups, respectively, and *N* is the sample size of the high (or low) responder group. Based on an empirical Bayes approach, in order to stabilize the variance of *d*(*i*) irrespective of the gene expression level, *s*_0_ was introduced in the denominator of Equation (1) as described in Tusher et al. [18]. However, using a constant *s*_0_ value as in Tusher et al, the variance of *d*(*i*) was not fully stabilized in our data, causing difficulty in detecting significantly differentially expressed genes in our application. To better reflect the characteristics of our dataset, we developed the following procedure: (1) calculate the relative difference, *d*(*i*), as in Equation (1) with varying *s*_0_ values. Specifically, *s*_0_ starts with a small constant value and decreases toward 0 as *s*(*i*) increases (see Additional file 1 for details); (2) for each of 1,000 permutations of the samples within each gene, calculate relative difference *d*_*p*_(*i*) to get the null distribution of the test statistic *d*(*i*); (3) to find significant changes in gene expression, get the corresponding *P* value of each observed relative difference *d*(*i*) based on the null distribution calculated in (2).

### Prediction using SVM

Radial-basis SVMs were used for training and predicting in the SVM classification and regression models. The performance of the models was evaluated as follows: the data were randomly split into 10 sets, with nine assigned as training set and the tenth as testing set. The model was then trained using the training set and applied to the testing set for prediction. This process was repeated 1,000 times and the prediction power of the model was estimated based on the 1,000 testing sets. The SVM function in the R package (kernlab) was used to implement the models with default parameter settings [36]. For the SVM the radial basis kernel was chosen due to its superior performance in the cross-validation results to other kernel functions such as linear, polynomial, or hyperbolic tangent kernel (Additional file 1: Figure S8). The classification performance was evaluated by ROC curve analysis and quantitated by AUC using the ROCR package in R [37].

### Data access

The gene expression data have been deposited in the Gene Expression Omnibus (GEO) under accession number GSE36868 and in Synapse [38] under accession number syn299510. The genotype data have been deposited in the database for genotypes and phenotypes (dbGaP, [39]) under accession number phs000481.

## Additional file

Additional file 1:
**Supplementary Figures S1 to S8 and Table S1 to S4.**

## Abbreviations

APOB: Apolipoprotein B
CAP: Cholesterol and Pharmacogenetics
eQTL: Expression quantitative trait loci
GWAS: Genome-wide association studies
HDLC: High-density lipoprotein cholesterol
LCLs: Lymphoblastoid cell lines
LDLC: Low density lipoprotein cholesterol
NMF: Non-negative matrix factorization
SNPs: Single nucleotide polymorphisms
SVM: Support vector machine
