# Supplementary material for: Prediction of LDL cholesterol response to statin using transcriptomic and genetic variation
Source: Genome Biol. 2014 Sep 30;15(9):460. doi: 10.1186/s13059-014-0460-9 (PMC4180544; doi:10.1186/s13059-014-0460-9)
Supplement: Additional file 1: — Supplementary Figures S1 to S8 and Table S1 to S4. [file 13059_2014_460_MOESM1_ESM.docx]

## Figure S1. Age-adjusted distribution of LDLC change after simvastatin treatment of 372 individuals from the CAP clinical trial. Twenty-six each of the highest and lowest responders were color-coded with red and blue, respectively.

## Figure S2. (a) Purity difference between true high *vs.* low responder groups and randomly selected group (the difference between the red and blue line in Figure 1A). (b) Entropy curves measuring the performance of NMF in clustering. The red line was calculated from the *N*/2 highest and *N*/2 lowest samples (*N* = 20, ⋅⋅⋅, 80) and the blue line was obtained from the *N* randomly selected samples from the entire set of samples.

**
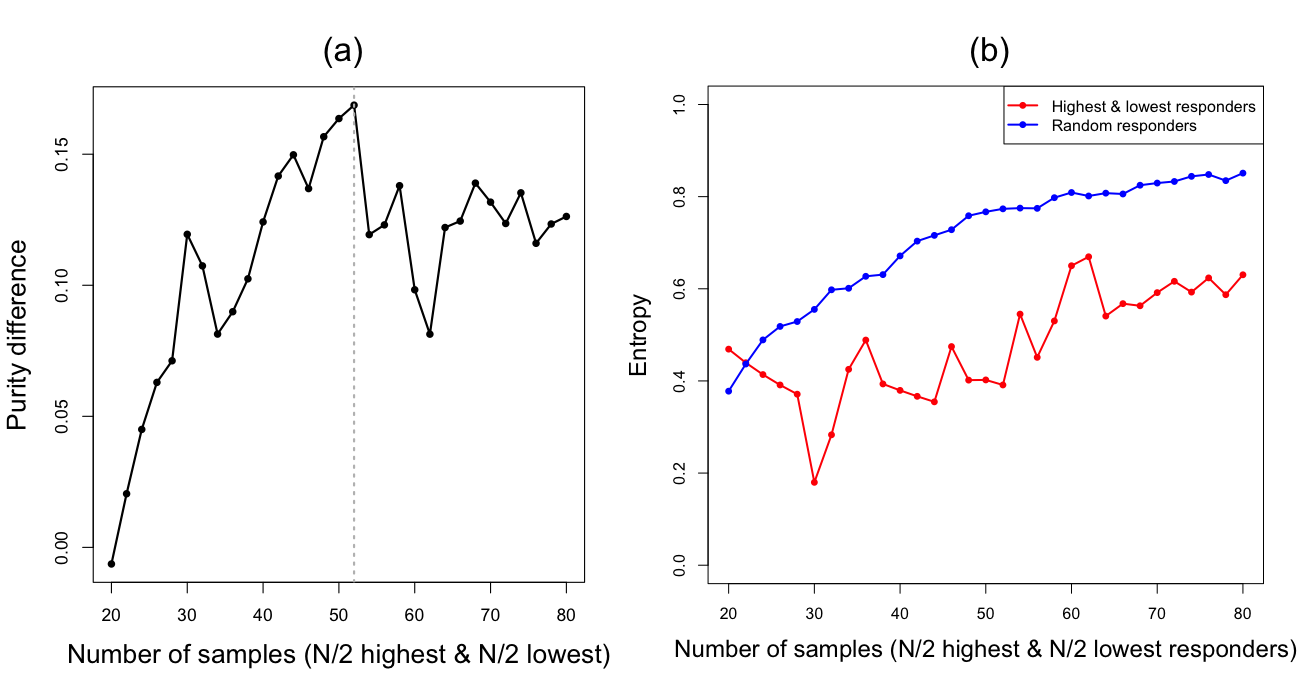
**

**Figure S3**. To decide the best number of genes from *SG*, (a) AUC calculated from the ROC curves and (b) explained variance in the statin-mediated LDLC change were calculated with expression levels of the 50, 100, 150, and 200 most significant genes. The goal of this analysis is not identifying individually significant genes after multiple testing adjustment, but selecting a set of genes that is most informative for the prediction of LDLC changes after statin treatment. Furthermore, there is a difference between statistical significance and biological significance. Some genes may not meet conventional criteria for statistical significance, but they may still carry unique information that is complementary to those significant genes for prediction purpose. Recognizing this, we selected our signature genes based on the prediction performance, and multiple testing adjustment did not affect this analysis.

## Figure S4. ROC curves from the prediction models incorporating various features or a combinations of (a) *SG*, (b) *SG_NO_*, (c) *SG* and 36 eQTLs, (d) 36 eQTLs, (e) *SG* and 7 GWAS SNPs, (f) 7 GWAS SNPs, and (g) all of the features.


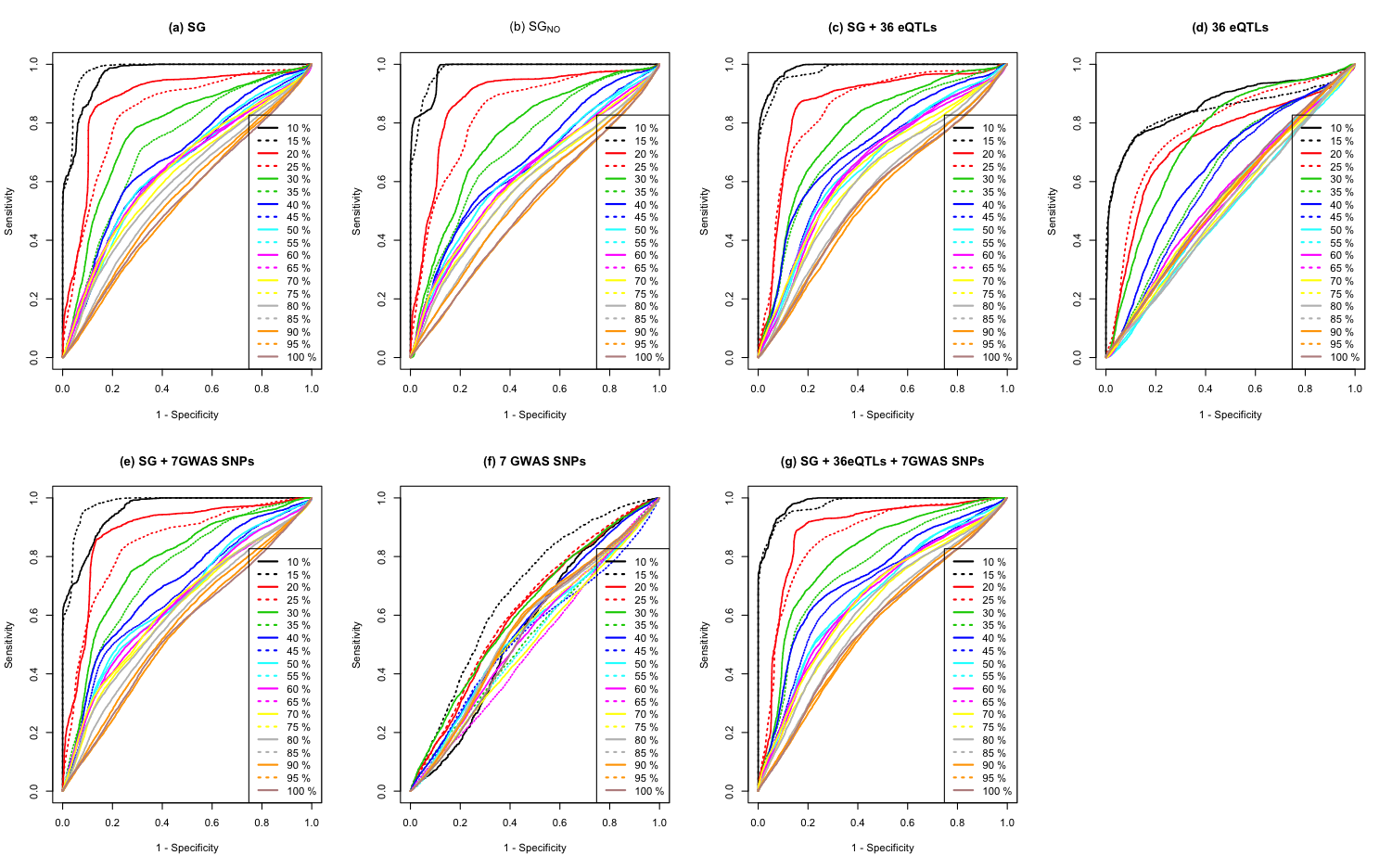


## Figure S5. AUC plots from SVM models each taking advantage of different features such as *SG* alone, *SG* with 22 GWAS SNPs (7 SNPs with *P* <5×10^-8^ and 15 SNPs with *P* <10^-6^) and 22 GWAS SNPs alone. For comparison, the results in Figure 3(c) are also provided.

**Figure S6.** Comparison of the distribution of LDLC change upon statin treatment between *CAP372* and *CAP212* using density plots (a) and box plots (b). To better compare both populations at tail, samples corresponding to 15% tail were color-coded with pink (high responders) and blue (low responders) in (c). While two high responders group showed similar levels of LDLC change, low responders from *CAP212* showed much more positive LDLC change values indicating these low responders were way more extreme than the one from *CAP372*.


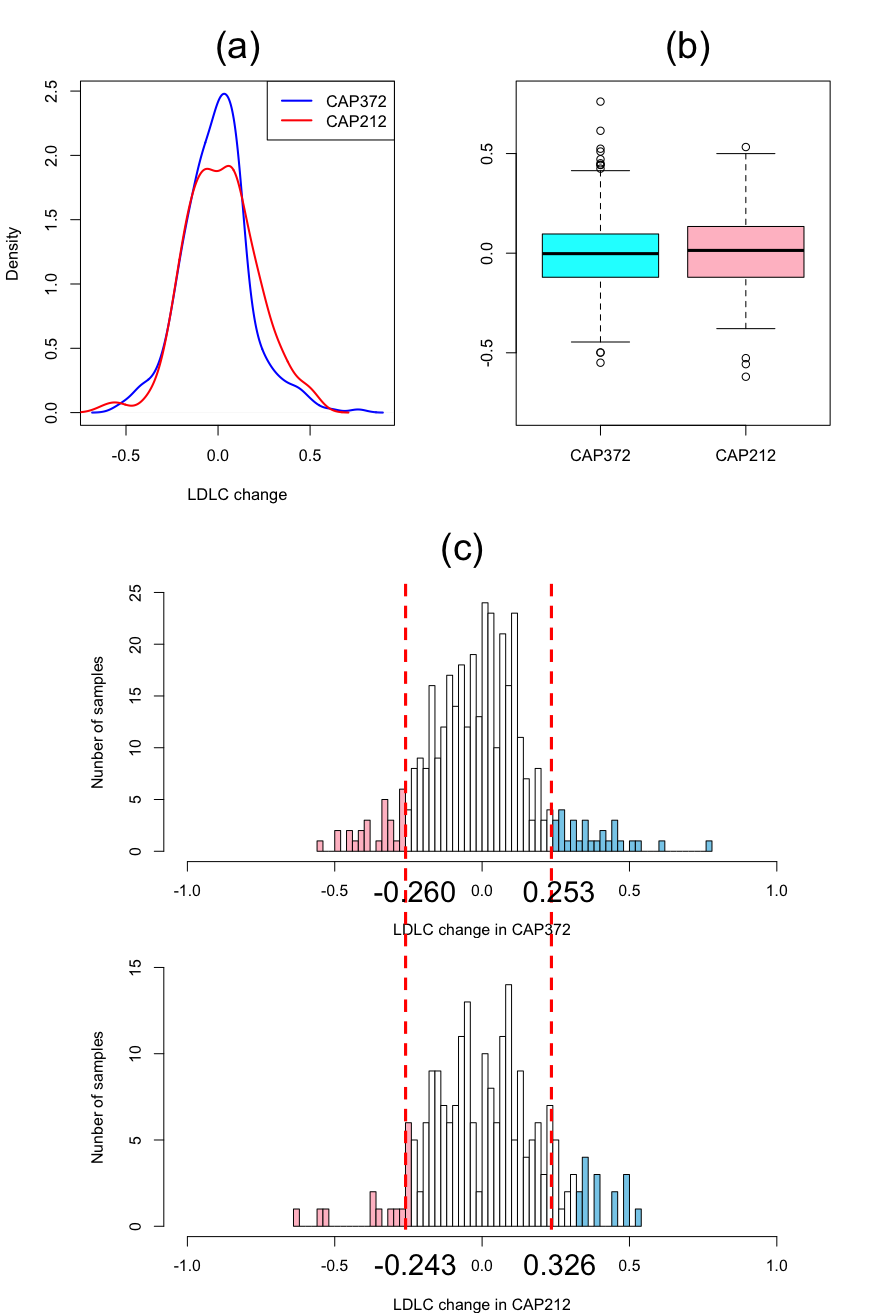


**Figure S7.** Comparison of the absolute and relative change in LDLC**.** While the absolute change (*b*-*a*) in LDLC is very highly correlated with the baseline values in the LDLC, the relative change in LDLC, calculated as log(b/a) is not correlated to baseline LDLC (*a* and *b* represent pre- and post-treatment LDLC value, respectively). Thus, testing for expression traits that are correlated with the absolute change (*b*-*a*), will primarily identify genes whose expression are correlated with baseline LDLC and not variation in statin response.


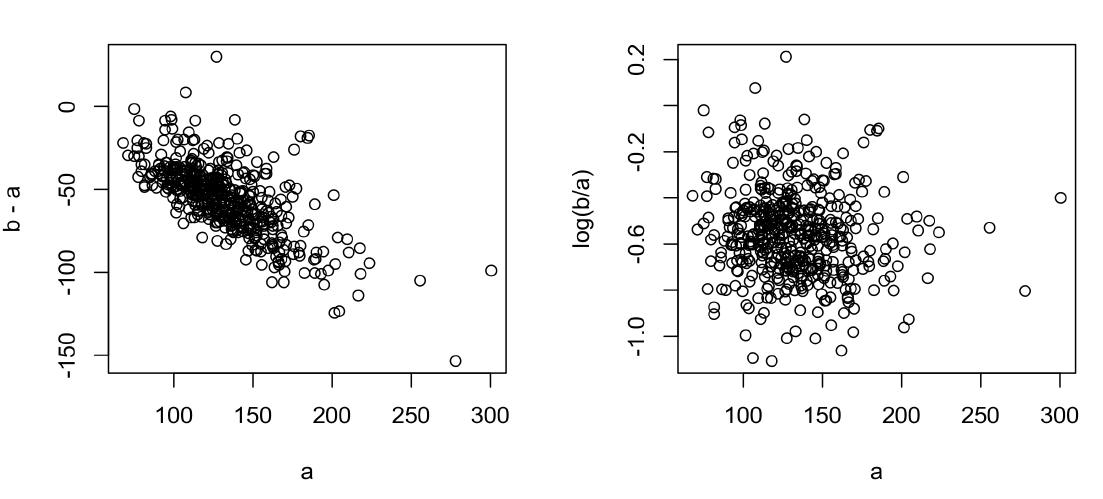


**Figure S8.** Comparison of four different kernel functions such as radial basis (RBF), polynomial, linear and sigmoid in a SVM classification model based on the expression levels of *SG*. In the comparison of the ROC curve (a) and the corresponding AUC values (b), radial basis kernel functions consistently outperformed others.

**
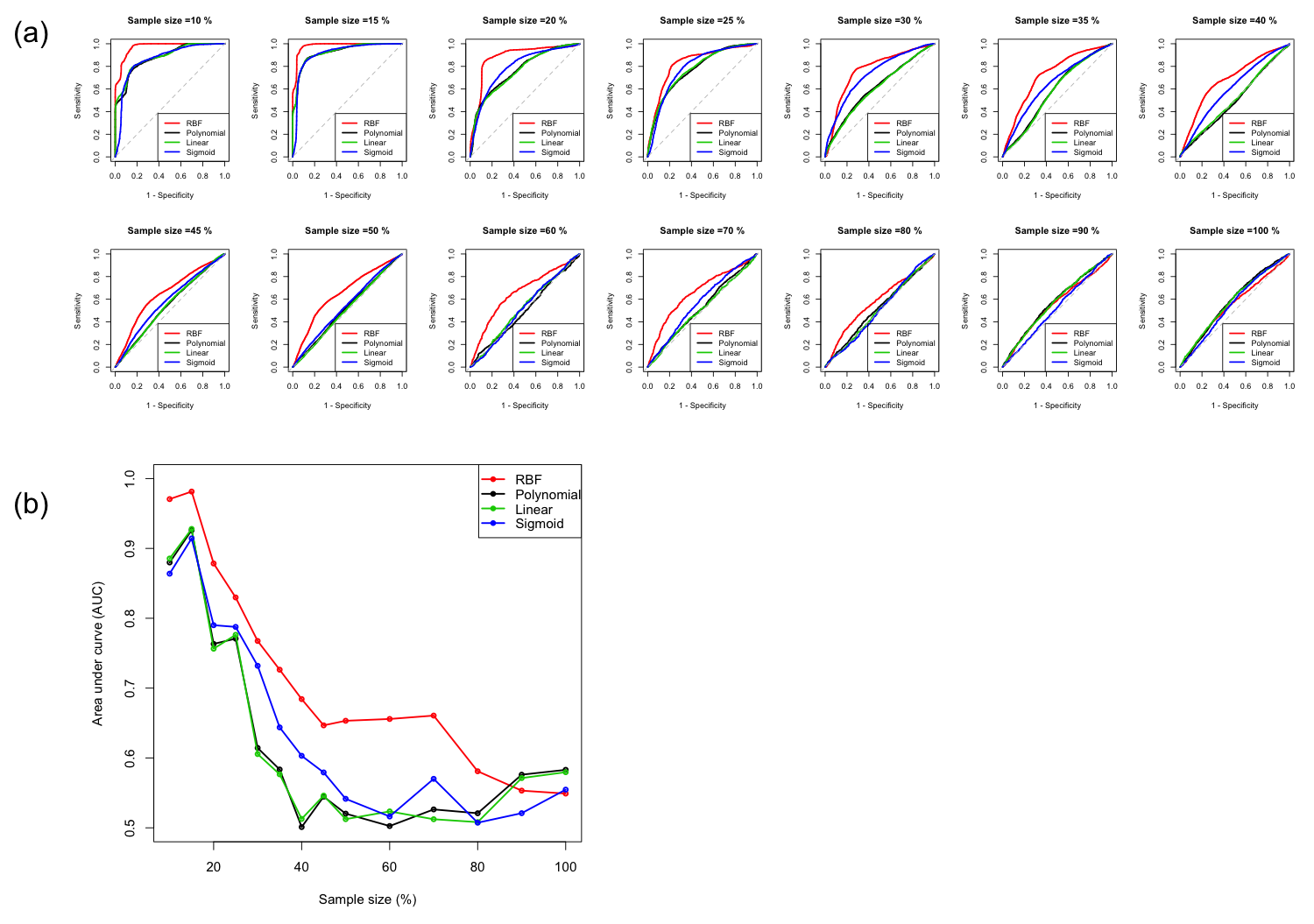
**

**Table S1.** List of 100 genes in *SG*. Genes with positive and negative *d*(*i*) were highly expressed in the high and low responders, respectively.

| **Gene** | ***d(i)*** | ***P* value** | **Gene** | ***d(i)*** | ***P* value** |
| --- | --- | --- | --- | --- | --- |
| *MFSD1* | 3.49 | 0 | *NFYC* | -3.52 | 0 |
| *SLC25A20* | 3.26 | 0 | *ZIK1* | -3.25 | 0 |
| *TTC33* | 3.12 | 0 | *GAGE4* | -3.24 | 0 |
| *MGAT2* | 3.03 | 0 | *TNFSF14* | -3.06 | 0 |
| *SAT2* | 2.94 | 0 | *ASF1B* | -3.02 | 0 |
| *ZNF398* | 2.86 | 0 | *IFIT3* | -3.01 | 3.3×10^-04^ |
| *C4orf41* | 2.76 | 0 | *ESPNL* | -2.99 | 3.3×10^-04^ |
| *CLK4* | 2.75 | 0 | *TMEM180* | -2.95 | 3.3×10^-04^ |
| *ITCH* | 2.75 | 0 | *SPOCK2* | -2.93 | 3.3×10^-04^ |
| *ACBD3* | 2.70 | 0 | *F12* | -2.92 | 3.3×10^-04^ |
| *SLC24A1* | 2.92 | 3.3×10^-04^ | *ICOS* | -2.87 | 6.7×10^-04^ |
| *SACM1L* | 2.83 | 3.3×10^-04^ | *MTA3* | -2.81 | 6.7×10^-04^ |
| *DSTN* | 2.73 | 3.3×10^-04^ | *PIP5K2A* | -2.79 | 1.0×10^-03^ |
| *PDPK1* | 2.61 | 3.3×10^-04^ | *SYNGR1* | -2.76 | 1.0×10^-03^ |
| *ERGIC2* | 2.54 | 3.3×10^-04^ | *RNF44* | -2.75 | 1.0×10^-03^ |
| *ZFR* | 2.86 | 6.7×10^-04^ | *TRAP1* | -2.70 | 1.0×10^-03^ |
| *RAP1B* | 2.71 | 6.7×10^-04^ | *SLMO1* | -2.64 | 1.0×10^-03^ |
| *RNF170* | 2.60 | 6.7×10^-04^ | *RIMBP2* | -2.58 | 1.3×10^-03^ |
| *KIAA1267* | 2.54 | 6.7×10^-04^ | *ZKSCAN2* | -2.54 | 1.7×10^-03^ |
| *C9orf43* | 2.49 | 6.7×10^-04^ | *IRF8* | -2.51 | 1.7×10^-03^ |
| *TMED10* | 2.29 | 6.7×10^-04^ | *HSBP1* | -2.50 | 1.7×10^-03^ |
| *NEDD9* | 2.21 | 6.7×10^-04^ | *PARP14* | -2.46 | 2.0×10^-03^ |
| *PRDM1* | 2.70 | 1.0×10^-03^ | *KCNJ14* | -2.37 | 2.0×10^-03^ |
| *TMED5* | 2.66 | 1.0×10^-03^ | *ITPKB* | -2.34 | 2.0×10^-03^ |
| *IER3IP1* | 2.37 | 1.0×10^-03^ | *NOB1* | -2.28 | 2.3×10^-03^ |
| *FBXW7* | 2.36 | 1.0×10^-03^ | *MBP* | -2.27 | 2.3×10^-03^ |
| *ARL5B* | 3.02 | 1.3×10^-03^ | *TNKS1BP1* | -2.17 | 2.7×10^-03^ |
| *ARF4* | 2.65 | 1.3×10^-03^ | *GDPD5* | -2.17 | 2.7×10^-03^ |
| *VPS54* | 2.57 | 1.3×10^-03^ | *KLHL35* | -2.15 | 3.0×10^-03^ |
| *ARL1* | 2.40 | 1.3×10^-03^ | *C19orf48* | -2.11 | 3.0×10^-03^ |
| *GPBP1* | 2.40 | 1.3×10^-03^ | *ABHD17AP2* | -2.00 | 3.0×10^-03^ |
| *CCDC90B* | 2.17 | 1.3×10^-03^ | *H2AFY* | -2.00 | 3.0×10^-03^ |
| *MTMR6* | 2.16 | 1.3×10^-03^ | *DDX41* | -1.93 | 3.0×10^-03^ |
| *SLC35A5* | 2.11 | 1.3×10^-03^ |  |  |  |
| *TMED7* | 2.38 | 1.7×10^-03^ |  |  |  |
| *ATG5* | 2.26 | 1.7×10^-03^ |  |  |  |
| *RAB40B* | 2.16 | 1.7×10^-03^ |  |  |  |
| *PDIK1L* | 2.11 | 1.7×10^-03^ |  |  |  |
| *CYP51A1* | 2.07 | 1.7×10^-03^ |  |  |  |
| *PPM1B* | 2.48 | 2.0×10^-03^ |  |  |  |
| *MIER3* | 2.47 | 2.0×10^-03^ |  |  |  |
| *CCDC41* | 2.42 | 2.0×10^-03^ |  |  |  |
| *PRDX5* | 2.08 | 2.0×10^-03^ |  |  |  |
| *SEC24A* | 2.60 | 2.3×10^-03^ |  |  |  |
| *RGL1* | 2.45 | 2.3×10^-03^ |  |  |  |
| *CHP1* | 2.18 | 2.3×10^-03^ |  |  |  |
| *MED23* | 2.84 | 2.7×10^-03^ |  |  |  |
| *ENTPD4* | 2.56 | 2.7×10^-03^ |  |  |  |
| *UFL1* | 2.45 | 2.7×10^-03^ |  |  |  |
| *TMEM183B* | 2.44 | 2.7×10^-03^ |  |  |  |
| *FAM91A1* | 2.37 | 2.7×10^-03^ |  |  |  |
| *ERLEC1* | 2.37 | 2.7×10^-03^ |  |  |  |
| *GOLPH3* | 2.34 | 2.7×10^-03^ |  |  |  |
| *CACNA2D2* | 2.29 | 2.7×10^-03^ |  |  |  |
| *NOL8* | 2.23 | 2.7×10^-03^ |  |  |  |
| *SAR1B* | 2.17 | 2.7×10^-03^ |  |  |  |
| *LOC401357* | 2.14 | 2.7×10^-03^ |  |  |  |
| *AGPAT4* | 2.10 | 2.7×10^-03^ |  |  |  |
| *ZNF197* | 2.99 | 3.0×10^-03^ |  |  |  |
| *COG6* | 2.74 | 3.0×10^-03^ |  |  |  |
| *CCDC50* | 2.65 | 3.0×10^-03^ |  |  |  |
| *NGLY1* | 2.45 | 3.0×10^-03^ |  |  |  |
| *C1orf63* | 2.40 | 3.0×10^-03^ |  |  |  |
| *PAPD4* | 2.30 | 3.0×10^-03^ |  |  |  |
| *GALK2* | 2.10 | 3.0×10^-03^ |  |  |  |
| *FOSB* | 2.45 | 3.3×10^-03^ |  |  |  |
| *FNDC3A* | 2.39 | 3.3×10^-03^ |  |  |  |

## Table S2. Datasets used to search the eQTL SNPs correlated with the identified signature genes in *SG*.

| **Tissue** | **Experiment method** | **Samples; Source** | **Authors** |
| --- | --- | --- | --- |
| LCLs | RNA-seq | 60; HAPMAP | Montgomery *et al.*, 2010 |
| Liver | Array | 427; HLC | Schadt *et al.*, 2008 |
| LCLs | Array | 210; HAPMAP | Stranger *et al.*, 2007 |
| LCLs | Array | 480; CAP | Mangravite *et al.*, 2013 |
| LCLs | Array | 1355; MRCA, MRCE | Liang *et al.*, 2013 |

## Table S3. SNPs associated with expression levels of *SG* genes.

| **SNP** | **Gene** | **Chromosome** | **Tissue** | ***P* value** | **Authors** |
| --- | --- | --- | --- | --- | --- |
| rs909685 | *SYNGR1* | 22 | LCL | 2.8×10^-73^ | Liang |
| rs1053454 | *PIP5K2A* | 10 | LCL | 8.3×10^-71^ | Mangravite |
| rs6557672 | *ENTPD4* | 8 | LCL | 8.9×10^-65^ | Mangravite |
| rs7994925 | *COG6* | 13 | LCL | 4.0×10^-38^ | Mangravite |
| rs11606662 | *GDPD5* | 11 | LCL | 8.7×10-^38^ | Mangravite |
| rs6034875 | *DSTN* | 20 | LCL | 8.3×10^-37^ | Liang |
| rs28395880 | *PRDX5* | 11 | LCL | 1.0×10^-36^ | Liang |
| rs2532332 | *KIAA1267* | 17 | Liver | 6.7×10^-36^ | Schadt |
| rs1055116 | *ARL5B* | 10 | LCL | 7.0×10^-34^ | Mangravite |
| rs4727018 | *ZNF398* | 7 | LCL | 2.8×10^-31^ | Liang |
| rs10874775 | *TMED5* | 1 | LCL | 2.5×10^-28^ | Mangravite |
| rs266128 | *C19orf48* | 19 | LCL | 8.7×10^-24^ | Mangravite |
| rs2731672 | *F12* | 5 | Liver | 1.1×10^-23^ | Schadt |
| rs6486572 | *RIMBP2* | 12 | LCL | 1.9×10^-23^ | Mangravite |
| rs1043641 | *ACBD3* | 1 | LCL | 6.3×10^-23^ | Liang |
| rs1641546 | *SAT2* | 17 | LCL | 2.8×10^-18^ | Mangravite |
| rs3859202 | *RAB40B* | 17 | LCL | 6.9×10^-18^ | Mangravite |
| rs9295813 | *NEDD9* | 6 | LCL | 9.7×10^-18^ | Stranger |
| rs10159774 | *IFIT3* | 10 | LCL | 2.4×10^-17^ | Mangravite |
| rs246344 | *SAR1B* | 5 | LCL | 4.4×10^-17^ | Mangravite |
| rs6809116 | *ZNF197* | 3 | LCL | 5.8×10^-17^ | Mangravite |
| rs58851861 | *GALK2* | 15 | LCL | 6.4×10^-17^ | Liang |
| rs1667901 | *MBP* | 18 | LCL | 1.0×10^-16^ | Mangravite |
| rs766968 | *SLC35A5* | 3 | LCL | 1.2×10^-16^ | Stranger |
| rs1077667 | *TNFSF14* | 19 | LCL | 3.7×10^-16^ | Mangravite |
| rs9578839 | *MTMR6* | 13 | LCL | 5.0×10^-16^ | Liang |
| rs1562339 | *ESPNL* | 2 | LCL | 7.0×10^-16^ | Mangravite |
| rs2516568 | *NOL8* | 9 | LCL | 7.5×10^-16^ | Liang |
| rs3087813 | *PAPD4* | 5 | Liver | 7.5×10^-13^ | Schadt |
| rs7953619 | *ERGIC2* | 12 | LCL | 5.6×10^-11^ | Stranger |
| rs2961669 | *CLK4* | 5 | LCL | 3.0×10^-10^ | Stranger |
| rs11117426 | *IRF8* | 16 | LCL | 2.6×10^-09^ | Mangravite |
| rs4744191 | *NGLY1* | 9 | Liver | 3.2×10^-09^ | Schadt |
| rs500300 | *SLMO1* | 18 | LCL | 8.9×10^-09^ | Mangravite |
| rs2712800 | *KLHL35* | 11 | Liver | 2.7×10^-08^ | Schadt |
| rs7833650 | *FAM91A1* | 8 | LCL | 5.0×10^-08^ | Mangravite |

## Table S4. List of seven GWAS SNPs known as genetic determinants of statin-induced LDLC reduction.

| **SNP** | **Gene** | **Chromosome** | ***P* value** |
| --- | --- | --- | --- |
| rs7412^a^ | *APOE* | 19 | 5.8×10^-19^ |
| rs445925^a^ | *APOE-APOC1*^b^ | 19 | 1.5×10^-17^ |
| rs1481012 | *ABCG2* | 4 | 1.7×10^-15^ |
| rs10455872 | *LPA* | 6 | 5.0×10^-15^ |
| rs2199936 | *ABCG2* | 4 | 2.1×10^-12^ |
| rs405509 | *APOE-TOMM40*^b^ | 19 | 3.4×10^-09^ |
| rs6857 | *PVRL2-TOMM40*^b^ | 19 | 7.4×10^-08^ |

# ^a^Only these two SNPs from the list have been found to be in linkage disequilibrium (LD) R^2^ = 0.588, in Caucasian population, from 1000 genome pilot 1.

# ^b^For SNPs located in the intergenic regions, the genes of nearby are shown.

**The summary statistics of the actual changes in LDL cholesterol level (mg/dl)**

Shown below are the summary statistics of the actual changes in LDL cholesterol level (mg/dl) from 942 participants of the Cholesterol and Pharmacogenetics (CAP) clinical trial. The corresponding graphical summary using a histogram and a boxplot is also provided for visualization.

| Min. | 1st Qu. | Median | Mean | 3rd Qu. | Max. | SD |
| --- | --- | --- | --- | --- | --- | --- |
| -153.50 | -67.88 | -53.50 | -54.12 | -40.50 | 30.00 | 22.40 |

**The effect of the subset size selected by NMF on the choice of the *SG***

Since an *N*=30 achieved the highest purity (Figure 1a), we compared prediction performance of signature genes derived from 30 *versus* 52 samples. In the regression model, signature genes derived from 30 and 52 samples explained a similar magnitude of variance, 12.9% and 12.3% respectively. However, in the classification model, signature genes derived from 30 samples performed much worse than from 52 samples (Figure a below) demonstrating the difficulty of reflecting the characteristics of extreme responders with too small number of samples. This finding supports our original selection of 52 samples as a reasonable choice.

To assess the effects of sample size on identification of signature genes, we compared the signature genes derived from 52 samples to those derived from 48, 50, 54, and 56 samples. As shown in Figures b and c (below), 72%, 79%, 82%, and 79% of the top ranked 100 genes derived from 48, 50, 54, and 56 samples were overlapped with our signature genes from 52 samples. Thus, although there is some effect of sample size on the choice of signature genes, it is not dramatic.

**
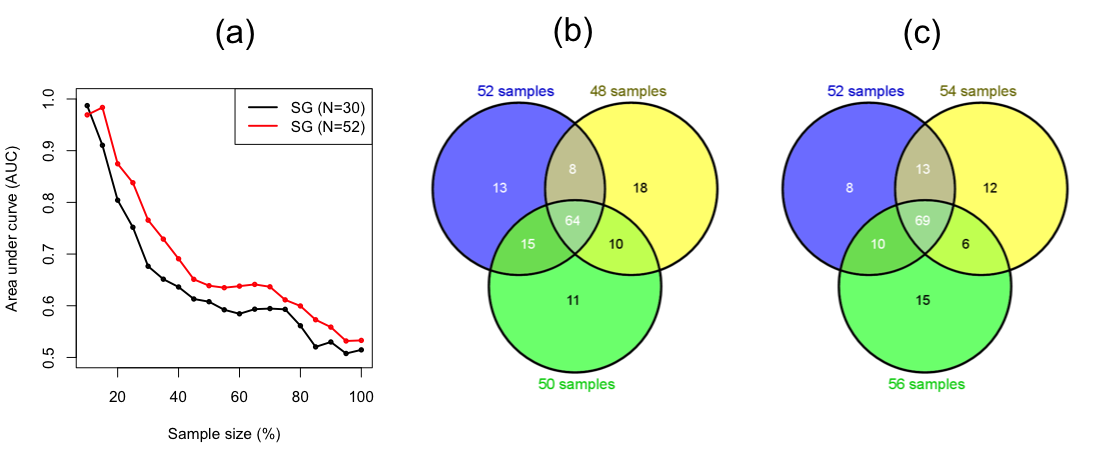
**

**More details of calculating varying *s_0_* values in Equation (1)**

As was discussed in Methods section, *s*_0_ was selected to minimize the coefficient of variation of *d*(*i*), which was computed as a function of *s*(*i*) in moving windows across data.

$d\left( i \right)=\frac{\overline{x}_{H}\left( i \right)-\overline{x}_{L}(i)}{s\left( i \right)+s_{0}}$ (1)

Specifically,

1. The *d*(*i*) were separated into approximately 100 groups. The 1% of the *d*(*i*) values with the smallest *s*(*i*) values were placed in the first group, the 1% of the *d*(*i*) values with the next smallest *s*(*i*) were placed in the second group, and so on.
2. The median absolute deviation (MAD) of the *d*(*i*) values was computed separately for each group.
3. The coefficient of variation (CV) of these 100 MAD values was computed.
4. For each of *s_0_* equal to the minimum of *s*(*i*), the 5th percentile of the *s*(*i*) values, the 10th percentile of the *s*(*i*) values,..., the 95th percentile of the *s*(*i*) values, steps (i) to (iii) were repeated for the varying *s_0_* values which were defined to start with *s_0_* and decreased toward 0 as *s*(*i*) increased.
5. The set of varying *s_0_* values that minimizes the CV of the 100 MAD values over candidate sets of varying s_0_ values described above was selected to replace *s_0_* in Equation (1).

**REFERENCES**

1. Montgomery SB, Sammeth M, Gutierrez-Arcelus M, Lach RP, Ingle C, Nisbett J, Guigo R, Dermitzakis ET: **Transcriptome genetics using second generation sequencing in a Caucasian population.** *Nature* 2010, **464:**773–777.
2. Schadt EE, Molony C, Chudin E, Hao K, Yang X, Lum PY, Kasarskis A, Zhang B, Wang S, Suver C, Zhu J, Millstein J, Sieberts S, Lamb J, GuhaThakurta D, Derry J, Storey JD, Avila-Campillo I, Kruger MJ, Johnson JM, Rohl CA, van Nas A, Mehrabian M, Drake TA, Lusis AJ, Smith RC, Guengerich FP, Strom SC, Schuetz E, Rushmore TH, *et al*: **Mapping the genetic architecture of gene expression in human liver.** *PLoS Biol* 2008, **6:**e107.
3. Stranger BE, Nica AC, Forrest MS, Dimas A, Bird CP, Beazley C, Ingle CE, Dunning M, Flicek P, Koller D, Montgomery S, Tavaré S, Deloukas P, Dermitzakis ET: **Population genomics of human gene expression.** *Nat Genet* 2007, **39:**1217–1224.
4. Mangravite LM, Engelhardt BE, Medina MW, Smith JD, Brown CD, Chasman DI, Mecham BH, Howie B, Shim H, Naidoo D, Feng Q, Rieder MJ, Chen YI, Rotter JI, Ridker PM, Hopewell JC, Parish S, Armitage J, Collins R, Wilke RA, Nickerson DA, Stephens M, Krauss RM: **A statin-dependent QTL for GATM expression is associated with statin-induced myopathy.** *Nature* 2013, **502**:377–380.
5. Liang L, Morar N, Dixon AL, Lathrop GM, Abecasis GR, Moffatt MF, Cookson WOC: **A cross-platform catalogue of 14,177 expression quantitative trait loci derived from lymphoblastoid cell lines.** *Genome Research* 2013, **23:**716–726.
